# Associations of Omega-3 Fatty Acid Supplement Use With Cardiovascular Disease Risks: Meta-analysis of 10 Trials Involving 77 917 Individuals

**DOI:** 10.1001/jamacardio.2017.5205

**Published:** 2018-01-31

**Authors:** Theingi Aung, Jim Halsey, Daan Kromhout, Hertzel C. Gerstein, Roberto Marchioli, Luigi Tavazzi, Johanna M. Geleijnse, Bernhard Rauch, Andrew Ness, Pilar Galan, Emily Y. Chew, Jackie Bosch, Rory Collins, Sarah Lewington, Jane Armitage, Robert Clarke

**Affiliations:** 1 Clinical Trial Service Unit and Epidemiological Studies Unit, Nuffield Department of Population Health, University of Oxford, Oxford, England; 2 Medical Research Council Population Health Research Unit, Nuffield Department of Population Health, University of Oxford, Oxford, England; 3 Department of Endocrinology, Royal Berkshire Hospital, Reading, England; 4 Department of Epidemiology, University of Groningen, Groningen, Netherlands; 5 Department of Medicine McMaster University, Hamilton Health Sciences, Hamilton, Ontario, Canada; 6 Population Health Research Institute, McMaster University, Hamilton Health Sciences, Hamilton, Ontario, Canada; 7 Cardiovascular Renal Metabolic Therapeutic Area, Medical Strategy and Science, Therapeutic Science and Strategy Unit, Quintiles, Milan, Italy; 8 Department of Cardiovascular Research, IRCCS Istituto di Ricerche Farmacologiche Mario Negri, Milan, Italy; 9 Maria Cecilia Hospital, GVM Care & Research, ES Health Science Foundation, Cotignola, Italy; 10 Division of Human Nutrition, Wageningen University, Wageningen, Netherlands; 11 Institut für Herzinfarktforschung Ludwigshafen, Ludwigshafen, Germany; 12 National Institute for Health Research, Bristol Biomedical Research Centre, University Hospitals Bristol National Health Service Foundation Trust, University of Bristol, Bristol, England; 13 Nutritional Epidemiology Research Team, Sorbonne Paris Cité Epidemiology and Biostatistics Research Center, Bobigny, France; 14 Division of Epidemiology and Clinical Applications, National Eye Institute, National Institutes of Health, Bethesda, Maryland; 15 School of Rehabilitation Science, McMaster University, Hamilton, Ontario, Canada

## Abstract

**Importance:**

Current guidelines advocate the use of marine-derived omega-3 fatty acids supplements for the prevention of coronary heart disease and major vascular events in people with prior coronary heart disease, but large trials of omega-3 fatty acids have produced conflicting results.

**Objective:**

To conduct a meta-analysis of all large trials assessing the associations of omega-3 fatty acid supplements with the risk of fatal and nonfatal coronary heart disease and major vascular events in the full study population and prespecified subgroups.

**Data Sources and Study Selection:**

This meta-analysis included randomized trials that involved at least 500 participants and a treatment duration of at least 1 year and that assessed associations of omega-3 fatty acids with the risk of vascular events.

**Data Extraction and Synthesis:**

Aggregated study-level data were obtained from 10 large randomized clinical trials. Rate ratios for each trial were synthesized using observed minus expected statistics and variances. Summary rate ratios were estimated by a fixed-effects meta-analysis using 95% confidence intervals for major diseases and 99% confidence intervals for all subgroups.

**Main Outcomes and Measures:**

The main outcomes included fatal coronary heart disease, nonfatal myocardial infarction, stroke, major vascular events, and all-cause mortality, as well as major vascular events in study population subgroups.

**Results:**

Of the 77 917 high-risk individuals participating in the 10 trials, 47 803 (61.4%) were men, and the mean age at entry was 64.0 years; the trials lasted a mean of 4.4 years. The associations of treatment with outcomes were assessed on 6273 coronary heart disease events (2695 coronary heart disease deaths and 2276 nonfatal myocardial infarctions) and 12 001 major vascular events. Randomization to omega-3 fatty acid supplementation (eicosapentaenoic acid dose range, 226-1800 mg/d) had no significant associations with coronary heart disease death (rate ratio [RR], 0.93; 99% CI, 0.83-1.03; *P* = .05), nonfatal myocardial infarction (RR, 0.97; 99% CI, 0.87-1.08; *P* = .43) or any coronary heart disease events (RR, 0.96; 95% CI, 0.90-1.01; *P* = .12). Neither did randomization to omega-3 fatty acid supplementation have any significant associations with major vascular events (RR, 0.97; 95% CI, 0.93-1.01; *P* = .10), overall or in any subgroups, including subgroups composed of persons with prior coronary heart disease, diabetes, lipid levels greater than a given cutoff level, or statin use.

**Conclusions and Relevance:**

This meta-analysis demonstrated that omega-3 fatty acids had no significant association with fatal or nonfatal coronary heart disease or any major vascular events. It provides no support for current recommendations for the use of such supplements in people with a history of coronary heart disease.

## Key Points

**Question:**

Does supplementation with marine-derived omega-3 fatty acids have any associations with reductions in fatal or nonfatal coronary heart disease in people at high risk of cardiovascular disease?

**Findings:**

This meta-analysis of 10 trials involving 77 917 participants demonstrated that supplementation with marine-derived omega-3 fatty acids for a mean of 4.4 years had no significant association with reductions in fatal or nonfatal coronary heart disease or any major vascular events.

**Meaning:**

The results provide no support for current recommendations to use omega-3 fatty acid supplements for the prevention of fatal coronary heart disease or any cardiovascular disease in people who have or at high risk of developing cardiovascular disease.

## Teaser

This meta-analysis of 10 large trials assessed the associations of omega-3 fatty acid supplements with the risk of fatal and nonfatal coronary heart disease and major vascular events in 77 917 high-risk individuals.

## Introduction

Observational studies in Western and Asian populations have reported that regular consumption of fish once or twice a week is associated with lower risks of death from coronary heart disease (CHD).^1,2^ These observations, together with the lower rates of CHD in populations that consumed large amount of foods rich in very-long-chain polyunsaturated fatty acids containing omega-3 fatty acids have prompted interest in assessing whether consumption of marine-derived very-long-chain omega-3 fatty acids (abbreviated “omega-3 FA” in this article) may be protective for CHD.^3^ These marine-derived omega-3 FAs include eicosapentaenoic acid (EPA) and docosahexanoic acid (DHA) found in fish and other seafood, but not alpha-linolenic acid, which is plant-derived.

The initial Diet and Reinfarction Trial-1 study^4^ examined the associations of consumption of oily fish twice or more per week with CHD risk in men who had had a myocardial infarction and reported that fish consumption was associated with a significant reduction in fatal CHD and all-cause mortality but had no association with nonfatal myocardial infarction (MI) recurrence.^4^ However, the subsequent Diet and Reinfarction Trial-2 study in men with angina reported that consumption of fish or fish oil supplements increased the risk of CHD death.^5^ Subsequently, several large trials have reported conflicting results of the associations of supplementation with omega-3 FA supplements vs placebo or untreated controls on fatal and nonfatal vascular events.^6,7,8,9,10,11,12,13,14,15,16^

Ten large randomized trials^6,7,8,9,10,11,12,13,14,15^ have been conducted comparing the associations of treatment with omega-3 FA supplementation vs placebo or no treatment for at least 12 months in populations with prior CHD, stroke, or high risk of cardiovascular disease (CVD). These trials have reported conflicting results for the associations of treatment with fatal CHD, nonfatal CHD, or other subtypes of CVD. The Gruppo Italiano per lo Studio della Sopravvivenza nell'Infarto Miocardico (GISSI)-Prevenzione trial,^6^ an open-label trial involving 11 323 recent survivors of MI, reported that patients who received supplementation with omega-3 FAs experienced a 10% reduced risk of major cardiovascular events compared with untreated controls. The Japan EPA Lipid Intervention Study (JELIS) trial, an open-label trial involving 18 645 participants with total cholesterol of 243.24 mg/dL (to convert to mmol/L, multiply by 0.0259) or greater, of whom only 20% with prior CHD, also reported^14^ that supplementation with fish oil was associated with a 19% reduction in major CHD events (95% CI, 5%-31%). None of the other large placebo-controlled trials reported any significant association with CHD or mortality. Hence, it is unclear whether the discrepant results reflect different associations of omega-3 FAs with CHD subtypes, different outcomes in primary vs secondary prevention of CHD, increasing use of statins with better control of lipid levels, or an artifact of chance or bias in open-label trials. Previous meta-analyses of these trials of omega-3 FA supplements^16,17,18^ appeared to suggest a significant beneficial association of omega-3 FAs with fatal CHD but not nonfatal CHD. However, these meta-analyses were constrained because they included trials of dietary advice to eat fish^17^ or excluded trials that did not include a placebo control.^18^

The Omega-3 Treatment Trialists’ Collaboration was established to conduct a collaborative meta-analysis based on aggregated study-level data obtained from the principal investigators of all large randomized clinical trials of omega-3 FA supplements for the prevention of cardiovascular disease, using a prespecified protocol and analysis plan. The aims of this meta-analysis were to assess the associations of supplementation with omega-3 FAs on (1) fatal CHD, nonfatal MI, stroke, major vascular events, and all-cause mortality and (2) major vascular events in prespecified subgroups.

## Methods

We performed a systematic search of randomized clinical trials in PubMed and Medline data sets, supplemented by manual hand-searching of reference lists from individual trials, review articles, or previous meta-analyses of omega-3 FAs and CVD (eFigure 1 in the Supplement). Search terms included “omega-3 FA,” “omega-3 polyunsaturated fat,” “fish oils,” and “ω-3 FA” and “cardiovascular disease” or “coronary heart disease” or “stroke” (eFigure 1 in the Supplement). The prespecified eligibility criteria were randomized clinical trials of marine-derived very-long-chain omega-3 FA supplements vs placebo or open-label control, with a sample size of at least 500 participants and a scheduled duration of treatment of at least 1 year. All eligible trials required use of supplements, but no minimum daily dose of EPA or DHA was specified. The prespecified end points included nonfatal MI; death caused by CHD; ischemic, hemorrhagic, and unclassified stroke; coronary or noncoronary arterial revascularization events; major vascular events (a composite of first occurrence of nonfatal MI or death caused by CHD; nonfatal or fatal stroke; or any revascularization procedure); and all-cause mortality. Deaths caused by CHD included sudden cardiac deaths, deaths due to ventricular arrhythmias, and heart failure in patients with CHD, MI, or deaths occurring after coronary revascularization or heart transplant.

All included trials were also assessed for risk of bias. Individual trials had approval from their respective institutional review boards, and all participants provided written informed consent. No additional ethical approval was required for this meta-analysis.

A protocol outlining the eligibility criteria, prespecified analyses, and plans for publication together with standardized data request forms were sent to the principal investigators of all eligible trials. The study used the PRISMA guidelines for the conduct of meta-analysis of randomized trials.^19^ Aggregated study-level (tabular) data were successfully obtained from 9 of the 10 trials (Table; eTable in the Supplement).^6,7,8,9,10,11,12,13,15^ The JELIS trial^14^ declined to participate in this collaboration, but the published results of the trial were sufficiently detailed to allow its inclusion in this study. Any discrepancies between data supplied and the published reports were clarified by contacting trial investigators.

**Table.** Characteristics of Included Trials

Study (Year)	Patients, No.	Dose of EPA/ DHA (mg/d)	Male, No. (%)	Mean Trial Duration, y	Mean (SD) Age, y	No. (%)			
						Prior CHD	Prior Stroke	Prior Diabetes	Statin Use
DOIT (2010)	563	1150/800	563 (100)	3	70 (3)	133 (23.6)	37 (6.6)	46 (8.2)	NA
AREDS-2 (2014)	4203	650/350	1816 (43.2)	4.5	74 (NA)	405 (9.7)	211 (5.0)	546 (13.0)	1866 (44.4)
SU.FOL.OM3 (2010)	2501	400/200	1987 (79.4)	4.7	61 (NA)	1863 (74.5)	638 (25.5)	440 (17.9)	2079 (83.1)
JELIS (2007)^a^^,^^b^	18 645	1800/NA	5859 (31.4)	4.6	61 (8)	NA	NA	3040 (16.3)	18 645 (100.0)
Alpha Omega (2010)	4837	226/150	3783 (78.2)	3.3	69 (6)	4837 (100.0)	345 (7.2)	1014 (21.0)	4122 (85.2)
OMEGA (2010)	3818	460/380	2841 (74.4)	1	64 (NA)	796 (22.5)	192 (5.5)	948 (27.0)	3566 (94.2)
R&P (2013)	12 505	500/500	7687 (61.5)	5	64 (NA)	Not stated (30)	594 (4.8)	7494 (59.9)	12 505 (100.0)
GISSI-HF (2008)	6975	850/950	5459 (78.3)	3.9	67 (11)	3614 (51.8)	346 (5.0)	1974 (28.3)	NA
ORIGIN (2012)	12 536	465/375	8150 (65.0)	6.2	64 (8)	8094 (64.6)	10 877 (86.8)	11 081 (88.4)	6739 (53.8)
GISSI-P^b^ (1999)	11 334	850/1700	9658 (85.2)	3.5	59 (11)	11 334 (100.0)	NA	2139 (18.9)	NA
Total	77 917	NA	47 803 (61.4)	4.4	64	31 076/ 46 767 (66.4)	13 240/ 47 938 (27.6)	28 722 (36.9)	49 522 (83.4)

Abbreviations: AREDS-2, Age-Related Eye Disease Study 2; DOIT, Diet and Omega-3 Intervention Trial; GISSI-HF, Gruppo Italiano per lo Studio della Sopravvivenza nell'Infarto Miocardico-Heart Failure; GISSI-P, Gruppo Italiano per lo Studio della Sopravvivenza nell'Infarto Miocardico-Prevenzione; JELIS, Japan Eicosapentaenoic Acid (EPA) Lipid Intervention Study; NA, not available; OMEGA, Effect of Omega 3-Fatty Acids on the Reduction of Sudden Cardiac Death After Myocardial Infarction; ORIGIN, Outcome Reduction With Initial Glargine Intervention; SU.FOL.OM3, Supplémentation en Folates et Omega-3; R&P, Risk and Prevention Study.

^a^ All trials used eicosapentaenoic acid and docosahexanoic acid supplements, with the exception of the JELIS trial (eicosapentaenoic acid only).

^b^ All trials were blind, placebo-controlled randomized clinical trials with the exception of JELIS and GISSI-P, which were open-label without placebo.

### Statistical Analysis

The association of treatment with outcomes in each trial was analyzed separately, and summary statistics were calculated for each trial. For each trial, we calculated the observed minus expected statistic (O−E) and its variance (V) from the number of patients who developed the relevant end point and the total number of patients in each treatment group, using standard formulas for 2 × 2 contingency tables. One O−E value from each trial was summed to produce a grand total (G), with variance (V) equal to the sum of their separate variances. The value exp(G/V) is Peto 1-step estimate of the rate ratio (RR), and its continuity-corrected 95% confidence interval is given by exp(G/V ± [0.5/V + (1.96/√V)]).^20^ Rate ratios are given with 95% CI for the overall results for major diseases and with 99% CI (which is calculated by replacing 1.96 in the formula above by 2.58) for the results of individual trials or subgroups of trials or subgroups of such major diseases. Heterogeneity between the different subgroups is assessed by first calculating S−(G^2^/V), where S is the sum of (O−E)^2^/V for each trial (or subgrouping), and then testing this statistic against a χ^2^ distribution with the degrees of freedom equal to 1 fewer than the number of subgroups. The meta-analysis was repeated after excluding the JELIS trial,^14^ since it tested EPA alone rather than the combination of EPA and DHA used in all other trials.^6,7,8,9,10,11,12,13,15^

Additional analyses of the primary outcomes assessed the associations of treatment with major vascular events in pre-defined subgroups, including age, sex, prior CHD, prior stroke, prior diabetes, blood lipids (total cholesterol, triglyceride, high-density lipoprotein cholesterol, and calculated or measured low-density lipoprotein cholesterol), prior use of statins, and trial design (open-label or blinded). In interpreting subgroup results, the chief emphasis was placed on the overall results, unless there was strong evidence of heterogeneity (*P* < .001). Sensitivity analyses compared the results of the Peto method with log-rank method in the 1 trial that had also provided individual participant data on all events.

## Results

### Characteristics of Individual Trials

Study level data were obtained on a total of the 10 trials^6,7,8,9,10,11,12,13,14,15^ that met the inclusion criteria. A total of 77 917 participants were involved, and trials ranged in size from 563 to 18 645 participants (Table; eTable in the Supplement). Of the 10 trials, 8 had a double-blind design and used a placebo control, and 2 trials had an open-label design.^6,14^ The risk of bias of the included trials was low, with exception of the 2 trials that did not use a placebo-treated control group^6,14^ (eFigure 2 in the Supplement).

Combinations of polyunsaturated fatty acid ethyl esters of EPA and DHA were used in all but 1 trial,^14^ which tested daily dose of 1800 mg EPA alone. The daily doses of EPA varied from 226 to 1800 mg/day, and DHA varied from 0 to 1700 mg/day. The mean duration of treatment in individual trials varied from 1.0 year to 6.2 years (weighted mean, 4.4 years).

Of the 77 917 participants, 47 803 (61.4%) were men, and the mean age at entry was 64 years. After accounting for missing data, about two-thirds of participants had a prior history of CHD (31 076/46 767; 66.4%), 13 240 of 47 938 (28%) had prior stroke, and 28 722 of the total 77 917 participants (37%) had prior diabetes. Among the 77 917 participants, there were a total of 12 001 major vascular events (15.4% of 77 917 participants), including 2276 incidents of nonfatal MI (2.9%), 2695 CHD deaths (3.5%), 1713 strokes (2.2%), and 6603 revascularization events (8.5%) during the study duration (eTable in the Supplement). Data were available on the association of treatment by prior use of statin therapy in 7 trials involving 49 522 participants.^8,10,11,12,14,15^

### Associations of Omega-3 Fatty Acid Use With CHD and Major Vascular Events

**Figure 1.**
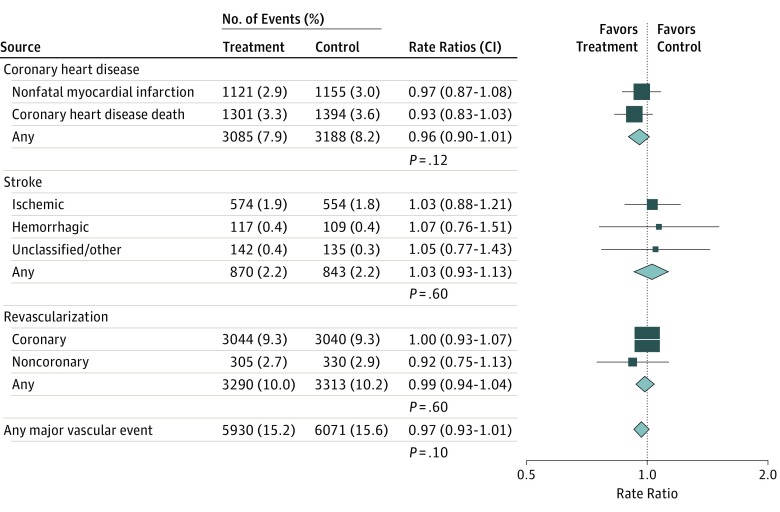
Associations of Omega-3 Fatty Acids With Major Vascular Events The number of events by allocated treatment are presented for individual trials and subgroups of trials; participants can contribute only once to subtotals and totals of major vascular events. Rate ratios for individual trials or subgroups of trials are indicated by squares and 99% CIs by horizontal lines. Overall totals and their 95% confidence intervals are represented by diamonds. The size of the squares and the diamonds are proportional to the statistical information conveyed.

**Figure 2.**
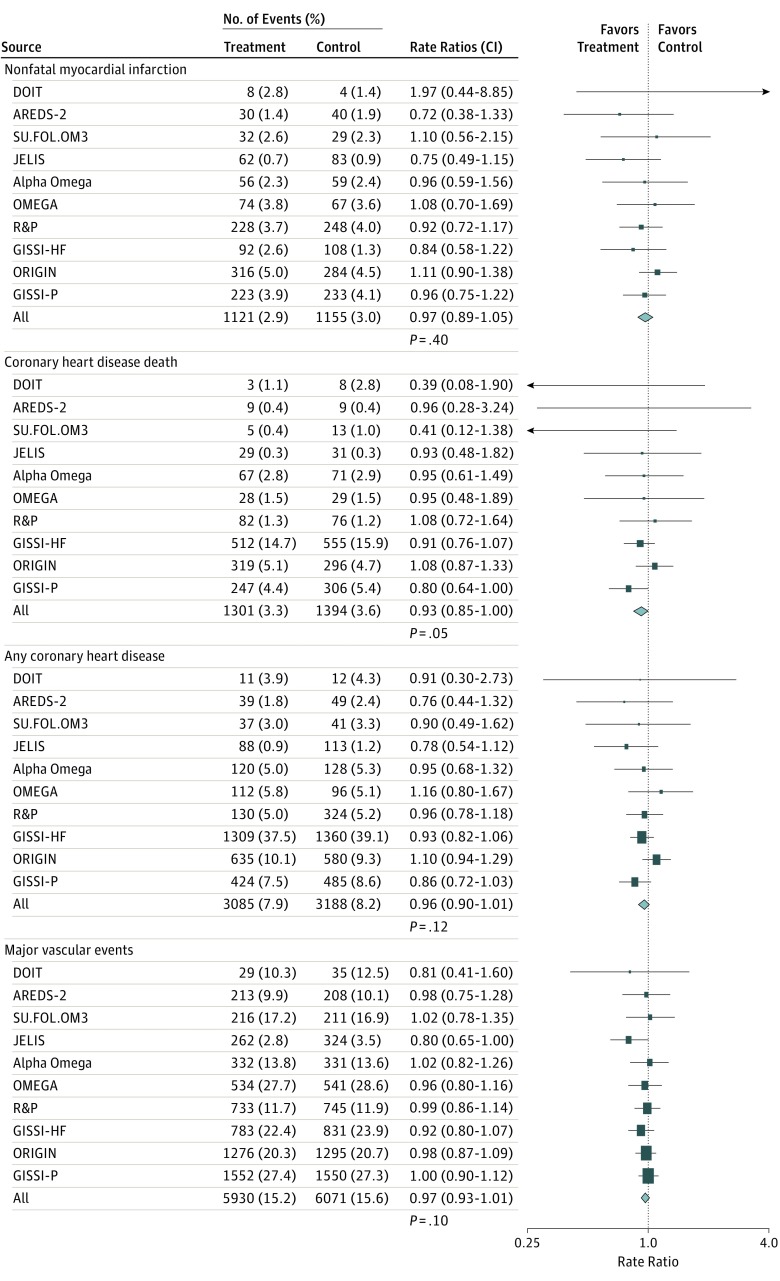
Associations of Omega-3 Fatty Acids With Subtypes of Coronary Heart Disease and Major Vascular Events, by Trial Symbols and conventions as in Figure 1. Study names are AREDS-2, Age-Related Eye Disease Study 2; DOIT, Diet and Omega-3 Intervention Trial; GISSI-HF, Gruppo Italiano per lo Studio della Sopravvivenza nell'Infarto Miocardico-Heart Failure; GISSI-P, Gruppo Italiano per lo Studio della Sopravvivenza nell'Infarto Miocardico-Prevenzione; JELIS, Japan Eicosapentaenoic Acid (EPA) Lipid Intervention Study; OMEGA, Effect of Omega 3-Fatty Acids on the Reduction of Sudden Cardiac Death After Myocardial Infarction; ORIGIN, Outcome Reduction With Initial Glargine Intervention; SU.FOL.OM3, Supplémentation en Folates et Omega-3; R&P, Risk and Prevention Study. Rate ratios for individual trials or subgroups of trials are indicated by squares and the 99% CIs by the horizontal lines. Overall totals and their 95% confidence intervals are represented by diamonds. Arrowheads indicate error bars that extend beyond the area shown. Heterogeneity between all trials (χ^2^_9_ in all cases) for nonfatal myocardial infarction, coronary heart disease death, any coronary heart disease, and major vascular events were 10.18 (*P* = .34), 12.3 (*P* = .20), 12.92 (*P* = .17), and 7.68 (*P* = .57), respectively.

Figure 1 shows that randomization to receive omega-3 FA supplementation had no significant association with the rate ratios (RRs) for any CHD event (RR, 0.96; 95% CI, 0.90-1.01; *P* = .12) and no significant association with RRs in subgroups of CHD events, including CHD death (RR, 0.93; 99% CI, 0.83-1.03; *P* = .05) and nonfatal myocardial infarction (RR, 0.97; 99% CI, 0.87-1.08; *P* = .40). Likewise, randomization of patients to an omega-3 FA supplementation regimen had no associations with the RRs for major vascular events (RR, 0.97; 95% CI, 0.93–1.01; *P* = .10), stroke (RR, 1.03; 95% CI, 0.93-1.13; *P* = .56), or revascularization events (RR, 0.99; 95% CI, 0.94-1.04; *P* = .61). This meta-analysis also showed no significant heterogeneity between the results of individual trials for nonfatal MI, CHD death, any CHD events, or all major vascular events (Figure 2). The association of omega-3 FA supplementation with major vascular events were unaltered after excluding the JELIS trial^14^ (odds ratio [OR], 0.98; 95% CI, 0.94-1.02; *P* = .30) (eFigure 3 in the Supplement). Additional sensitivity analyses in 1 trial^12^ that compared the results of the Peto method (O−E statistic) with the log-rank method demonstrated that analysis of individual participant and study-level data yielded identical results for association of omega-3 FA supplementation with major vascular events (eFigure 4 in the Supplement).

### Associations of Omega-3 Fatty Acid Use With Major Vascular Events in Prespecified Subgroups

**Figure 3.**
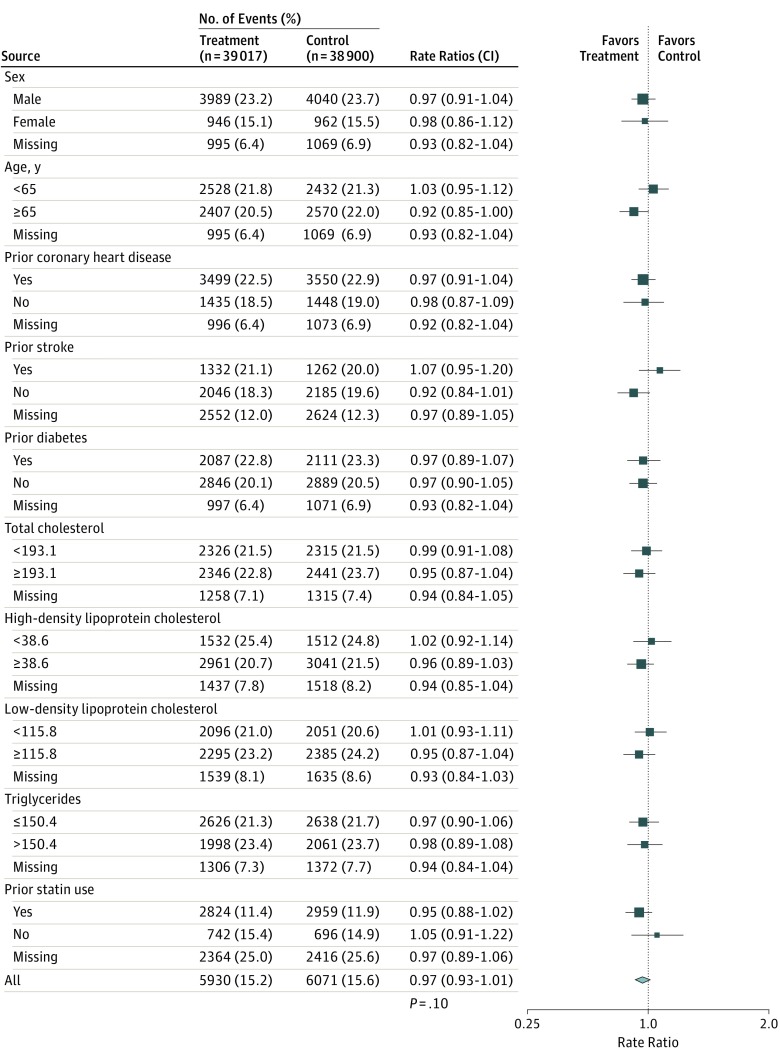
Associations of Omega-3 Fatty Acids With Major Vascular Events, in Prespecified Subgroups Symbols and conventions as in Figure 1. Total cholesterol, high-density lipoprotein cholesterol, low-density lipoprotein cholesterol, and triglycerides were measured in mg/dL (to convert cholesterol to mmol/L, multiply by 0.0259; triglycerides, multiply by 0.0113). Heterogeneity between all trials (χ^2^_1_ in all cases) was 0.04 (*P* = .84) for sex, 5.59 (*P* = .02) for age, 0.0 (*P* = .96) for prior coronary heart disease, 7.03 (*P* = .01) for prior stroke, 0.0 (*P* > .99) for prior diabetes, 0.87 (*P* = .35) for total cholesterol, 1.56 (*P* = .21) for high-density lipoprotein cholesterol, 1.8 (*P* = .18) for low-density lipoprotein cholesterol, 0.02 (*P* = .89) for triglycerides, and 2.55 (*P* = .11) for prior statin use.

Figure 3 shows that after adjustment for multiple testing, randomization of patients to study arms involving supplementation by omega-3 FAs had no significant association with major vascular events in any of the prespecified subgroups, including those defined by sex, history of CHD, history of diabetes, pretreatment levels of total cholesterol, high-density lipoprotein cholesterol levels, low-density lipoprotein cholesterol levels, triglyceride levels, or prior use of statin therapy. However, there was some evidence of heterogeneity in the associations of omega-3 FAs with major vascular events by age (unadjusted *P* = .02) and by history of stroke (*P* = .06), respectively. While it was not possible to assess the associations of treatment with race, the results were unaltered after exclusion of the JELIS trial,^14^ which was conducted in a Japanese population only (eFigure 3 in the Supplement).

### Associations of Omega-3 Fatty Acid Use With CHD Events by Study Design

**Figure 4.**
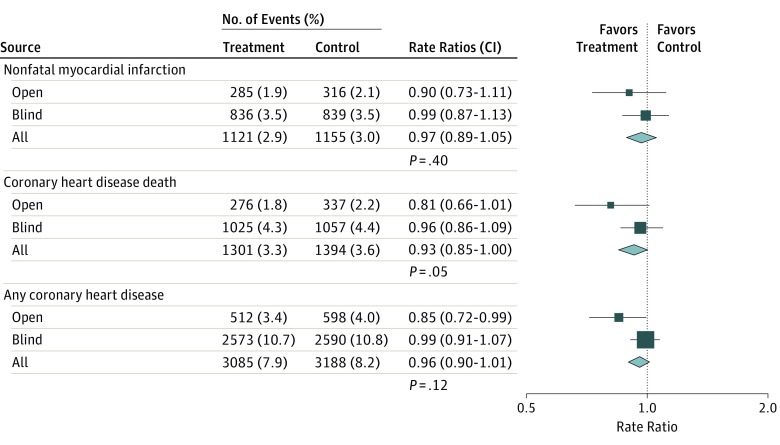
Associations of Omega-3 Fatty Acids With Fatal and Nonfatal Vascular Events, by Trial Design Symbols and conventions as in Figure 1. Heterogeneity between trial designs (χ^2^_1_ in all cases) was 1.05 (*P* = .31) for nonfatal myocardial infarction, 3.26 (*P* = .07) for coronary heart disease death, and 4.81 (*P* = .03) for any coronary heart disease.

Figure 4 demonstrates that randomization of patients to receive omega-3 FAs had no significant association with their experience of nonfatal MI, CHD death, or overall CHD in trials that used either an open-label and blind design. However, there was some evidence of heterogeneity between the results of open-label trials vs blind trials for all participants with CHD (open-label trials: RR, 0.85; 99% CI, 0.72-0.99; *P* = .01; blinded trials: RR, 0.99; 99% CI, 0.91-1.07; *P* = .69; heterogeneity *P* = 0.03), but not for either fatal CHD or nonfatal MI, respectively. Overall, the results of this meta-analysis demonstrated no significant association of supplementation with omega-3 FAs for a mean duration of 4.4 years with the risk of fatal CHD, nonfatal MI, any CHD, or any major vascular events in the full study population and in all relevant subgroups.

### Associations of Omega-3 Fatty Acid Use With All-Cause Mortality

Randomization to omega-3 FA intervention had no significant association with RRs of all-cause mortality (RR, 0.96; 95% CI, 0.92-1.01; *P* = .16). Further information is presented in eFigure 5 in the Supplement.

## Discussion

This meta-analysis of 10 randomized clinical trials, involving 77 917 participants, demonstrated that randomization to trial arms with omega-3 FA supplementation for a mean of 4.4 years had no significant effect on either of fatal CHD, nonfatal MI, stroke, revascularization events, or any major vascular events. Importantly, this meta-analysis also demonstrated no significant effect on major vascular events in any particular subgroups, including prior vascular disease, diabetes, lipid levels, or statin use. Likewise, the present meta-analysis showed no significant association of omega-3 FA supplementation with all-cause mortality or cancer (data not shown). Moreover, the overall results were unaltered after exclusion of the JELIS trial,^14^ which tested the effects of EPA alone rather than EPA and DHA combined.

The chief strength of this study was the availability of study-level data extracted by the trial principal investigators for all prespecified outcomes in this meta-analysis (with the exception of the JELIS trial,^14^ in which the published data were used). The inclusion criteria and vascular disease outcomes differed from previous meta-analyses of the published results.^16,17,18^ The present meta-analysis had a low risk of selection bias or confounding because it did not include trials testing the effects of dietary advice to eat fish nor trials that were either too small or insufficient in treatment duration. In contrast with previous meta-analyses, the present meta-analysis also examined effects of supplementation with omega-3 FA supplementation in prespecified subgroups of major vascular events by history of disease, history of diabetes, lipid levels, or statin use.

The reasons for the discrepant results of the previous trials of omega-3 FA supplementation on fatal and nonfatal CHD events are unclear. In contrast with the null findings for most trials, the GISSI-Prevenzione trial^6^ reported a 14% reduction in major vascular events, chiefly owing to an 11% reduction in cardiac deaths. But the JELIS trial reported a 19% (95% CI, 5%-31%) reduction in major CHD events (albeit based on only 586 events), chiefly owing to a reduction in nonfatal CHD events.^14^ It is unclear whether differences in inclusion criteria for prior diseases, concomitant use of statins, or other secondary prevention treatments may explain some of the conflicting results of individual trials.

For example, previous reports had suggested that the effects of omega-3 FA use may vary by patients’ prior use of statin medications.^21,22^ The Alpha Omega trial reported that use of low-dose omega-3 FAs reduced the risk of major vascular events in patients with prior MI who were not treated with statin medications.^22^ However, the present meta-analysis demonstrated no heterogeneity in the effects of omega-3 FA supplementation on CHD death or nonfatal MI between the individual trials and reported no differences in the effects of omega-3 FAs on major vascular events by subgroups of those with or without prior cardiovascular disease or diabetes; those with lipid levels less than or greater than specified cutoff points; or those who had histories of statin therapy. The results of the present meta-analysis were also unaltered by the exclusion of the JELIS trial,^14^ in which all participants were also treated with statin medications.

The present meta-analysis reported weak evidence of heterogeneity between the results of open-label vs blind trials for any CHD. This may reflect reporting bias, chance, or greater compliance in the open-label trials than in the blinded trials.

Previous meta-analyses of omega-3 FA trials,^16,17,18^ which were limited by being incomplete, including trials of dietary advice to increase fish consumption,^16,17^ or failure to distinguish the effects on a wide range of subtypes of CVD.^16,17,18,23,24^ In contrast, the present meta-analysis demonstrated that omega-3 FA supplementation had no significant effect on fatal CHD or any other CVD subtypes. Moreover, the conclusions of the present meta-analysis are consistent with those of a 2016 report^24^ for the US Agency for Healthcare Research and Quality that also involved study-level data from the same 10 large trials for prevention of major vascular events, and concluded that omega-3 FA supplementation had no association with the risk of major vascular events, all-cause mortality, sudden cardiac death, or revascularization. In contrast with this report, the present article was able to assess effects on a wide range of subtypes of CVD and on major vascular events in all relevant subgroups.^24^

### Limitations

This meta-analysis had several limitations. The protocol did not prespecify assessment of the effects of treatment by smoking status or by site-specific cancer incidence. An additional limitation of this meta-analysis involved the use of aggregated study-level data rather than individual-level data. A meta-analysis of individual participant data may have a greater chance of detecting effects of omega-3 FA supplements on subtypes of fatal CHD events (ie, sudden death or ventricular arrhythmias) in a wider range of subgroups. However, the overall null results of the present meta-analysis, which assesses effects on a wide range of prespecified CVD subtypes, provides little encouragement for such an approach. In addition, sensitivity analyses using data from 1 trial^12^ that also provided data on all individual participants indicated identical effect estimates and 99% CI for analyses using both O-E and log-rank methods.

The 95% CI in the present meta-analysis of 10 trials, involving 77 917 high-risk individuals, 12 001 major vascular events, and 6273 CHD events, cannot exclude a 7% lower risk of major vascular events and a 10% lower risk of CHD associated with omega-3 FA supplements. Several ongoing large randomized trials involving a total of 54 354 additional participants (A Study of Cardiovascular Events in Diabetes [ASCEND],^25^ n = 15 480; VITamin D and OmegA-3 TriaL [VITAL],^26^ n = 25 874; STatin Residual risk reduction with EpaNova in hiGh CV risk patienTs with Hypertriglyceridemia [STRENGTH],^27^ n = 13 000 and Reduction of Cardiovascular Events With EPA–Intervention Trial [REDUCE-IT], n = 8000) will provide additional evidence about the associations of omega-3 FA supplementation with the risk of major vascular events, any CHD, and subtypes of fatal and nonfatal CHD. Importantly, the STRENGTH^27^ and REDUCE-IT trials will test the effects on major vascular events of much higher doses of omega-3 FAs (3-4 g/d), which will lower plasma levels of triglycerides.

## Conclusions

The 2016 European Society of Cardiology and European Atherosclerosis Society guidelines for prevention of cardiovascular disease^28^ indicated that it is debatable whether omega-3 FAs may exert a protective effect, and the 2016 guidelines on the management of dyslipidaemia^29^ indicated that more evidence on the efficacy of omega-3 FA supplements for prevention of clinical outcomes is needed to justify their prescription. In contrast, the American Heart Association recommended^30^ that the use of omega-3 FAs for prevention of CHD is probably justified in individuals with prior CHD and those with heart failure and reduced ejection fractions. However, the results of the present meta-analysis provide no support for the recommendations to use approximately 1 g/d of omega-3 FAs in individuals with a history of CHD for the prevention of fatal CHD, nonfatal MI, or any other vascular events. The results of the ongoing trials are needed to assess if higher doses of omega-3 FAs (3-4 g/d) may have significant effects on risk of major vascular events.

## Notes

Supplement. **eTable.** Distribution of events by trial **eFigure 1.** Screening and selection of included trials **eFigure 2.** Funnel plots for subtypes of CHD and for major vascular events **eFigure 3.** Effects of omega-3 fatty acids on subtypes of CHD and on major vascular events, by trial excluding JELIS **eFigure 4.** Effects of omega-3 fatty acids on risk of non-fatal myocardial infarction and stroke in SU.FOL.OM3, by analysis method **eFigure 5. **Effects of omega-3 fatty acids on total mortality, by trial

## References

[1] Kromhout D, Bosschieter EB, de Lezenne Coulander C The inverse relation between fish consumption and 20-year mortality from coronary heart disease. N Engl J Med. 1985;312(19):1205-1209. 3990713 10.1056/NEJM198505093121901

[2] Zheng J, Huang T, Yu Y, Hu X, Yang B, Li D Fish consumption and CHD mortality: an updated meta-analysis of seventeen cohort studies. Public Health Nutr. 2012;15(4):725-737. 21914258 10.1017/S1368980011002254

[3] Kromhout D, Yasuda S, Geleijnse JM, Shimokawa H Fish oil and omega-3 fatty acids in cardiovascular disease: do they really work? Eur Heart J. 2012;33(4):436-443. 21933782 10.1093/eurheartj/ehr362 PMC3279313

[4] Burr ML, Fehily AM, Gilbert JF, Effects of changes in fat, fish, and fibre intakes on death and myocardial reinfarction: Diet and Reinfarction Trial (DART). Lancet. 1989;2(8666):757-761. 2571009 10.1016/s0140-6736(89)90828-3

[5] Burr ML, Ashfield-Watt PA, Dunstan FD, Lack of benefit of dietary advice to men with angina: results of a controlled trial. Eur J Clin Nutr. 2003;57(2):193-200. 12571649 10.1038/sj.ejcn.1601539

[6] GISSI-Prevenzione Investigators (Gruppo Italiano per lo Studio della Sopravvivenza nell'Infarto miocardico) Dietary supplementation with n-3 polyunsaturated fatty acids and vitamin E after myocardial infarction: results of the GISSI-Prevenzione trial. Lancet. 1999;354(9177):447-455. 10465168

[7] Tavazzi L, Maggioni AP, Marchioli R, ; Gissi-HF Investigators Effect of n-3 polyunsaturated fatty acids in patients with chronic heart failure (the GISSI-HF trial): a randomised, double-blind, placebo-controlled trial. Lancet. 2008;372(9645):1223-1230. 18757090 10.1016/S0140-6736(08)61239-8

[8] Kromhout D, Giltay EJ, Geleijnse JM; Alpha Omega Trial Group N-3 fatty acids and cardiovascular events after myocardial infarction. N Engl J Med. 2010;363(21):2015-2026. 20929341 10.1056/NEJMoa1003603

[9] Einvik G, Klemsdal TO, Sandvik L, Hjerkinn EM A randomized clinical trial on N-3 polyunsaturated fatty acids supplementation and all-cause mortality in elderly men at high cardiovascular risk. Eur J Cardiovasc Prev Rehabil. 2010;17(5):588-592. 20389249 10.1097/HJR.0b013e328339cc70

[10] Bosch J, Gerstein HC, Dagenais GR, ; ORIGIN Trial Investigators N-3 fatty acids and cardiovascular outcomes in patients with dysglycemia. N Engl J Med. 2012;367(4):309-318. 22686415 10.1056/NEJMoa1203859

[11] Rauch B, Schiele R, Schneider S, ; OMEGA Study Group OMEGA, a randomized, placebo-controlled trial to test the effect of highly purified omega-3 fatty acids on top of modern guideline-adjusted therapy after myocardial infarction. Circulation. 2010;122(21):2152-2159. 21060071 10.1161/CIRCULATIONAHA.110.948562

[12] Galan P, Kesse-Guyot E, Czernichow S, Briancon S, Blacher J, Hercberg S; SU.FOL.OM3 Collaborative Group Effects of B vitamins and omega 3 fatty acids on cardiovascular diseases: a randomised placebo controlled trial. BMJ. 2010;341:c6273. 21115589 10.1136/bmj.c6273 PMC2993045

[13] Risk and Prevention Study Collaborative Group N-3 fatty acids in patients with multiple cardiovascular risk factors. Eur J Prev Cardiol. 2016;23(9):947-955.26525065

[14] Yokoyama M, Origasa H, Matsuzaki M, ; Japan EPA lipid intervention study (JELIS) Investigators Effects of eicosapentaenoic acid on major coronary events in hypercholesterolaemic patients (JELIS): a randomised open-label, blinded endpoint analysis. Lancet. 2007;369(9567):1090-1098. 17398308 10.1016/S0140-6736(07)60527-3

[15] Bonds DE, Harrington M, Worrall BB, ; Writing Group for the AREDS2 Research Group Effect of long-chain ω-3 fatty acids and lutein + zeaxanthin supplements on cardiovascular outcomes: results of the Age-Related Eye Disease Study 2 (AREDS2) randomized clinical trial. JAMA Intern Med. 2014;174(5):763-771. 24638908 10.1001/jamainternmed.2014.328

[16] Bucher HC, Hengstler P, Schindler C, Meier G N-3 polyunsaturated fatty acids in coronary heart disease: a meta-analysis of randomized controlled trials. Am J Med. 2002;112(4):298-304. 11893369 10.1016/s0002-9343(01)01114-7

[17] Rizos EC, Ntzani EE, Bika E, Kostapanos MS, Elisaf MS Association between omega-3 fatty acid supplementation and risk of major cardiovascular disease events: a systematic review and meta-analysis. JAMA. 2012;308(10):1024-1033. 22968891 10.1001/2012.jama.11374

[18] Kwak SM, Myung SK, Lee YJ, Seo HG; Korean Meta-analysis Study Group Efficacy of omega-3 fatty acid supplements (eicosapentaenoic acid and docosahexaenoic acid) in the secondary prevention of cardiovascular disease: a meta-analysis of randomized, double-blind, placebo-controlled trials. Arch Intern Med. 2012;172(9):686-694. 22493407 10.1001/archinternmed.2012.262

[19] Liberati A, Altman DG, Tetzlaff J, The PRISMA statement for reporting systematic reviews and meta-analyses of studies that evaluate health care interventions: explanation and elaboration. PLoS Med. 2009;6(7):e1000100. 19621070 10.1371/journal.pmed.1000100 PMC2707010

[20] Baigent C, Peto R, Gray R, Parish S, Collins R Large-scale randomized evidence: trials and meta-analyses of trials In: Warrell DA, Cox TM, Firth JD, eds. Oxford Textbook of Medicine. 5th ed Oxford, England: Oxford University Press; 2010:31-45.

[21] Saravanan P, Davidson NC, Schmidt EB, Calder PC Cardiovascular effects of marine omega-3 fatty acids. Lancet. 2010;376(9740):540-550. 20638121 10.1016/S0140-6736(10)60445-X

[22] Eussen SR, Geleijnse JM, Giltay EJ, Rompelberg CJ, Klungel OH, Kromhout D Effects of n-3 fatty acids on major cardiovascular events in statin users and non-users with a history of myocardial infarction. Eur Heart J. 2012;33(13):1582-1588. 22301766 10.1093/eurheartj/ehr499 PMC3388014

[23] Hooper L, Thompson RL, Harrison RA, Omega 3 fatty acids for prevention and treatment of cardiovascular disease. Cochrane Database Syst Rev. 2004;18(4):CD003177. 15495044 10.1002/14651858.CD003177.pub2 PMC4170890

[24] Agency for Heathcare Research and Quality Omega-3 fatty acids and cardiovascular disease: an updated systemative review; evidence report/technology assessment no. 223. https://effectivehealthcare.ahrq.gov/sites/default/files/related_files/fatty-acids-cardiovascular-disease_executive.pdf. Accessed December 19, 2017.

[25] Aung T, Haynes R, Barton J, ; ASCEND Study Collaborative Group Cost-effective recruitment methods for a large randomised trial in people with diabetes: a study of cardiovascular events in diabetes (ASCEND). Trials. 2016;17(1):286. 27296091 10.1186/s13063-016-1354-9 PMC4907276

[26] Manson JE, Bassuk SS, Lee IM, The VITamin D and OmegA-3 TriaL (VITAL): rationale and design of a large randomized controlled trial of vitamin D and marine omega-3 fatty acid supplements for the primary prevention of cancer and cardiovascular disease. Contemp Clin Trials. 2012;33(1):159-171. 21986389 10.1016/j.cct.2011.09.009 PMC3253961

[27] US National Library of Medicine Outcomes study to assess STatin Residual risk reduction with EpaNova in hiGh CV risk patienTs with Hypertriglyceridemia (STRENGTH), 2014 https://clinicaltrials.gov/ct2/show/NCT02104817. Accessed December 15, 2017.

[28] Piepolo MF, Hoes AW, Agewal S, 2016 guidelines on cardiovascular disease prevention in clinical practice. Eur Heart J. 2016;37(29):2315-2381. 27222591 10.1093/eurheartj/ehw106 PMC4986030

[29] Catapano AL, Graham I, De Backer G, ; Authors/Task Force Members; Additional Contributor 2016 ESC/EAS guidelines for the management of dyslipidaemias. Eur Heart J. 2016;37(39):2999-3058. 27567407 10.1093/eurheartj/ehw272

[30] Siscovick DS, Barringer TA, Fretts AM, ; American Heart Association Nutrition Committee of the Council on Lifestyle and Cardiometabolic Health; Council on Epidemiology and Prevention; Council on Cardiovascular Disease in the Young; Council on Cardiovascular and Stroke Nursing; Council on Clinical Cardiology Omega-3 polyunsaturated fatty acid (fish oil) supplementation and the prevention of clinical cardiovascular disease: a science advisory from the American Heart Association. Circulation. 2017;135(15):e867-e884. 28289069 10.1161/CIR.0000000000000482 PMC6903779

