# Supplementary material for: Associations of Omega-3 Fatty Acid Supplement Use With Cardiovascular Disease Risks: Meta-analysis of 10 Trials Involving 77 917 Individuals
Source: JAMA Cardiol. 2018 Jan 31;3(3):14–22. doi: 10.1001/jamacardio.2017.5205 (PMC5885893; doi:10.1001/jamacardio.2017.5205)
Supplement: Supplement. — eTable. Distribution of events by trial eFigure 1. Screening and selection of included trials eFigure 2. Funnel plots for subtypes of CHD and for major vascular events eFigure 3. Effects of omega-3 fatty acids on subtypes of CHD and on major vascular events, by trial excluding JELIS eFigure 4. Effects of omega-3 fatty acids on risk of non-fatal myocardial infarction and stroke in SU.FOL.OM3, by analysis method eFigure 5. Effects of omega-3 fatty acids on total mortality, by trial [file jamacardiol-3-14-s001.pdf]

Supplementary Online Content

Aung T, Halsey J, Kromhout D, et al. Associations of omega-3 fatty acid supplement use with cardiovascular disease risks: meta-analysis of 10 trials involving 77 917 individuals. Published online January 31, 2018. *JAMA Cardiol*. doi:10.1001/jamacardio.2017.5205

eTable 1. Distribution of events by trial
eFigure 1. Screening and selection of included trials
eFigure 2. Funnel plots for subtypes of CHD and for major vascular events
eFigure 3. Effects of omega-3 fatty acids on subtypes of CHD and on major vascular events, by trial excluding JELIS
eFigure 4. Effects of omega-3 fatty acids on risk of non-fatal myocardial infarction and stroke in SU.FOL.OM3, by analysis method
eFigure 5. Effects of omega-3 fatty acids on total mortality, by trial

This supplementary material has been provided by the authors to give readers additional information about their work.

© 2018 Aung T et al. *JAMA Cardiol.*

Supplementary Material for "Omega-3 fatty acids and risk of cardiovascular disease: meta-analysis of 10 trials involving 77,900 individuals"

Table of Contents

e-Table 1: Distribution of events by trial

eFigure 1: Screening and selection of included trials

eFigure 2: Funnel plots for subtypes of CHD and for major vascular events

e-Figure 3: Effects of omega-3 fatty acids on subtypes of CHD and on major vascular events, by trial excluding JELIS

e-Figure 4: Effects of omega-3 fatty acids on risk of non-fatal myocardial infarction and stroke in SU.FOL.OM3, by analysis method

e-Figure 5: Effects of omega-3 fatty acids on total mortality, by trial

The authors do hereby declare that all illustrations and figures in the manuscript are entirely original and do not require reprint permission.

© 2018 Aung T et al. JAMA Cardiol.

eTable 1: Distribution of events by trial

Trial	Number randomised	Non-fatal MI	CHD death	Any CHD	Stroke	Revascularisation	MVE
DOIT (2010)	563	12	11	23	17	24	64
AREDS-2 (2014)	4203	70	18	88	84	117	421
SU-FOL-OM3 (2010)	2501	61	18	78	67	351	427
JELIS (2007)	18645	145	60	201	328	413	586
ALPHA OMEGA (2010)	4837	115	138	248	101	408	663
OMEGA (2010)	3818	141	57	208	35	975	1075
R&P (2013)	12505	476	158	634	37	-	1478
GISSI-HF (2008)	6975	200	1067	2669	225	268	1614
ORIGIN (2012)	12536	600	615	1215	650	1762	2571
GISSI-P (1999)	11334	456	553	909	169	2285	3102
All	77917	2276	2695	6273	1713	6603	12001

© 2018 Aung T et al. JAMA Cardiol.

eFigure 1: Screening and selection of included trials

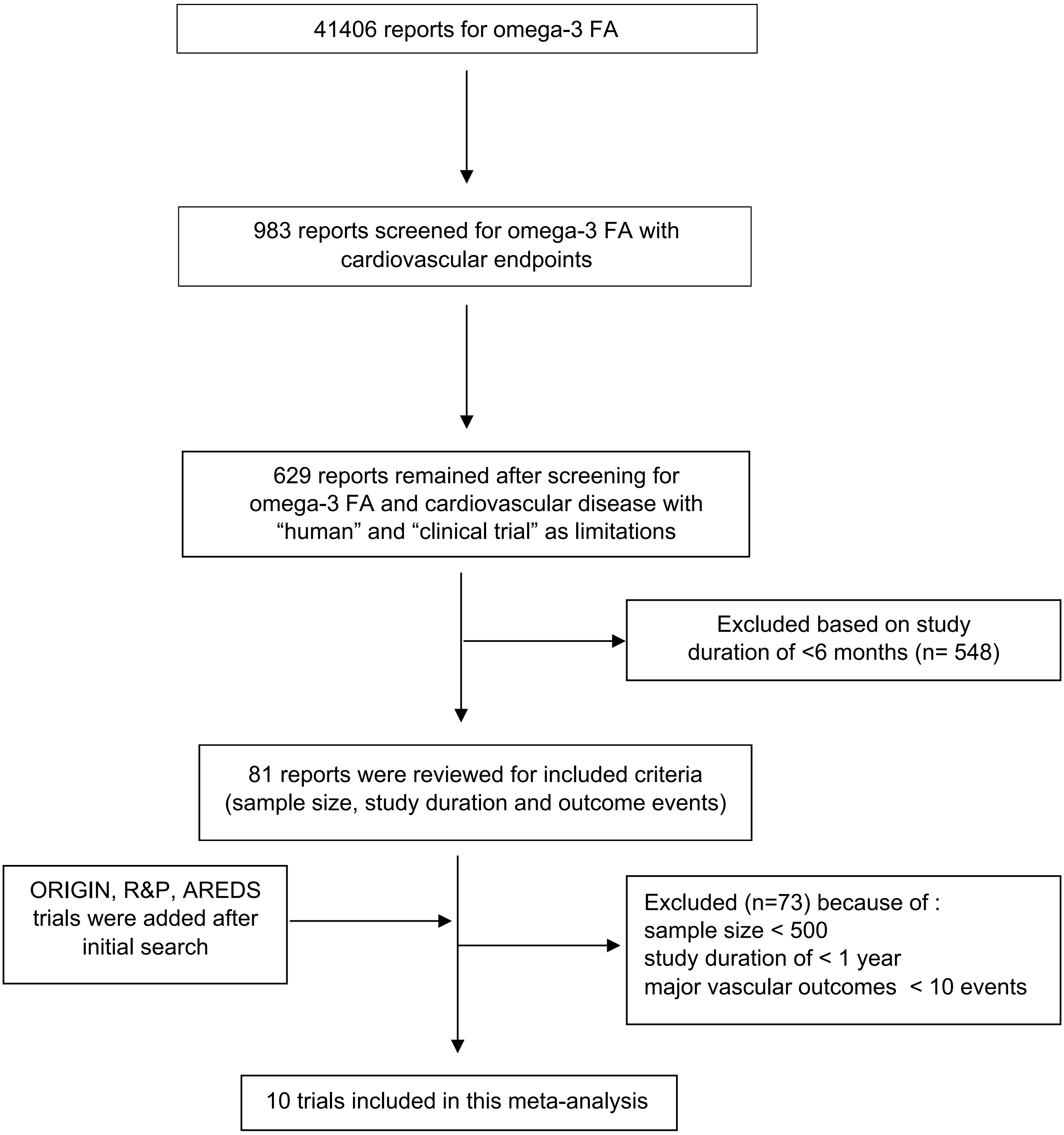

© 2018 Aung T et al. JAMA Cardiol.

eFigure 2: Funnel plots for subtypes of CHD and for major vascular events

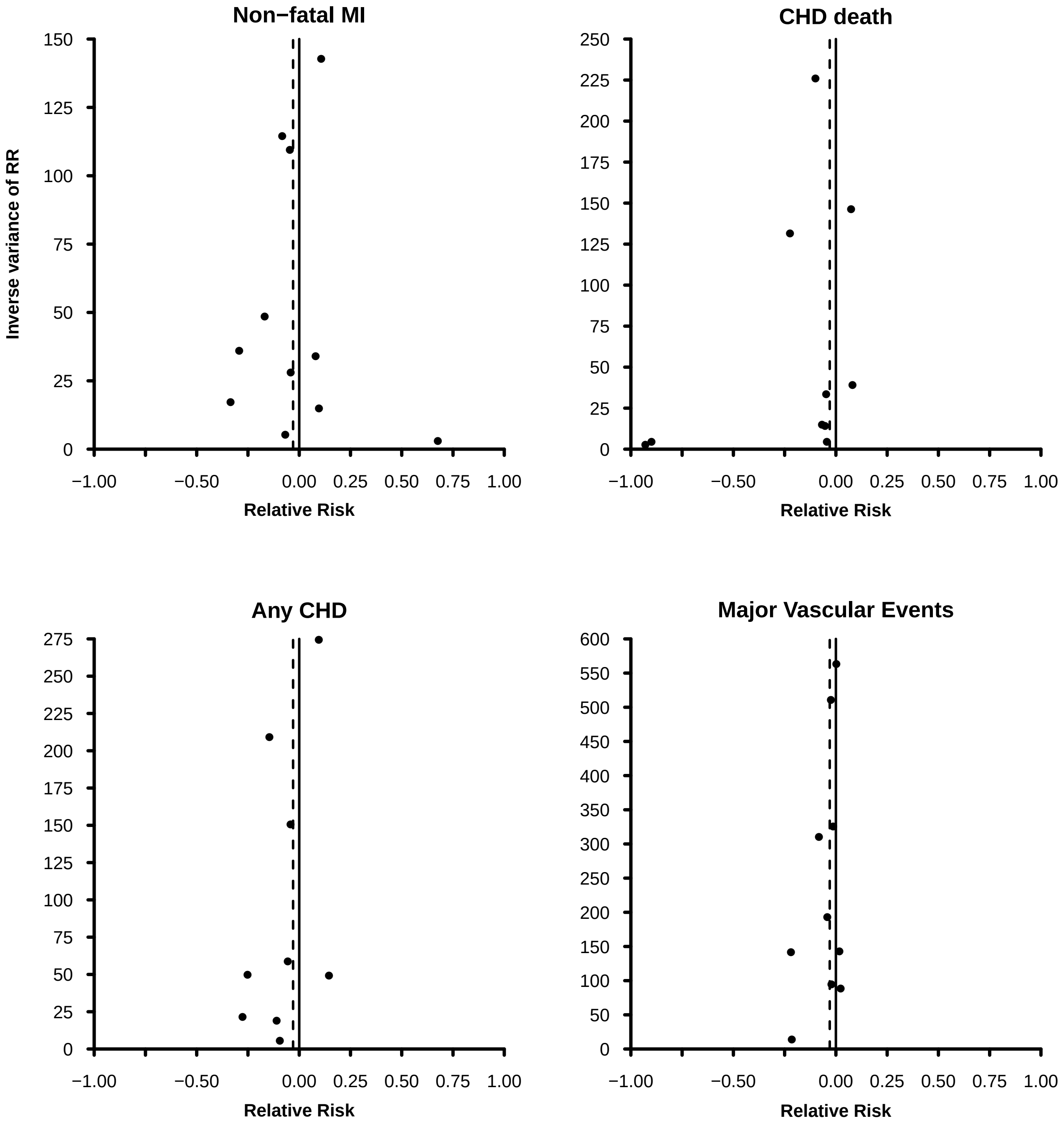

© 2018 Aung T et al. JAMA Cardiol.

eFigure 3: Effects of omega−3 fatty acids on subtypes of CHD and on major vascular events, by trial excluding JELIS

	Events (%) Treatment	Events (%) Control	RR (CI)
**Non−fatal MI**			
DOIT	8 (2.8)	4 (1.4)	1.97 (0.44−8.85)
AREDS−2	30 (1.4)	40 (1.9)	0.72 (0.38−1.33)
SU−FOL−OM3	32 (2.6)	29 (2.3)	1.10 (0.56−2.15)
ALPHA OMEGA	56 (2.3)	59 (2.4)	0.96 (0.59−1.56)
OMEGA	74 (3.8)	67 (3.6)	1.08 (0.70−1.69)
R&P	228 (3.7)	248 (4.0)	0.92 (0.72−1.17)
GISSI−HF	92 (2.6)	108 (3.1)	0.84 (0.58−1.22)
ORIGIN	316 (5.0)	284 (4.5)	1.11 (0.90−1.38)
GISSI−P	223 (3.9)	233 (4.1)	0.96 (0.75−1.22)
ALL	1059 (3.6)	1072 (3.6)	0.98 (0.90−1.07)
Heterogeneity: $\chi^2_8 = 7.6$; p=0.47			*p = 0.7*
**CHD death**			
DOIT	3 (1.1)	8 (2.8)	0.39 (0.08−1.90)
AREDS−2	9 (0.4)	9 (0.4)	0.96 (0.28−3.24)
SU−FOL−OM3	5 (0.4)	13 (1.0)	0.41 (0.12−1.38)
ALPHA OMEGA	67 (2.8)	71 (2.9)	0.95 (0.61−1.49)
OMEGA	28 (1.5)	29 (1.5)	0.95 (0.48−1.89)
R&P	82 (1.3)	76 (1.2)	1.08 (0.72−1.64)
GISSI−HF	512 (14.7)	555 (15.9)	0.91 (0.76−1.07)
ORIGIN	319 (5.1)	296 (4.7)	1.08 (0.87−1.33)
GISSI−P	247 (4.4)	306 (5.4)	0.80 (0.64−1.00)
ALL	1272 (4.3)	1363 (4.6)	0.92 (0.85−1.00)
Heterogeneity: $\chi^2_8 = 12.3$; p=0.14			*p = 0.06*
**Any CHD**			
DOIT	11 (3.9)	12 (4.3)	0.91 (0.30−2.73)
AREDS−2	39 (1.8)	49 (2.4)	0.76 (0.44−1.32)
SU−FOL−OM3	37 (3.0)	41 (3.3)	0.90 (0.49−1.62)
ALPHA OMEGA	120 (5.0)	128 (5.3)	0.95 (0.68−1.32)
OMEGA	112 (5.8)	96 (5.1)	1.16 (0.80−1.67)
R&P	310 (5.0)	324 (5.2)	0.96 (0.78−1.18)
GISSI−HF	1309 (37.5)	1360 (39.1)	0.93 (0.82−1.06)
ORIGIN	635 (10.1)	580 (9.3)	1.10 (0.94−1.29)
GISSI−P	424 (7.5)	485 (8.6)	0.86 (0.72−1.03)
ALL	2997 (10.1)	3075 (10.4)	0.96 (0.91−1.02)
Heterogeneity: $\chi^2_8 = 10.69$; p=0.22			*p = 0.2*
**Major Vascular Events**			
DOIT	29 (10.3)	35 (12.5)	0.81 (0.41−1.60)
AREDS−2	213 (9.9)	208 (10.1)	0.98 (0.75−1.28)
SU−FOL−OM3	216 (17.2)	211 (16.9)	1.02 (0.78−1.35)
ALPHA OMEGA	332 (13.8)	331 (13.6)	1.02 (0.82−1.26)
OMEGA	534 (27.7)	541 (28.6)	0.96 (0.80−1.16)
R&P	733 (11.7)	745 (11.9)	0.99 (0.86−1.14)
GISSI−HF	783 (22.4)	831 (23.9)	0.92 (0.80−1.07)
ORIGIN	1276 (20.3)	1295 (20.7)	0.98 (0.87−1.09)
GISSI−P	1552 (27.4)	1550 (27.3)	1.00 (0.90−1.12)
ALL	5668 (19.1)	5747 (19.4)	0.98 (0.94−1.02)
Heterogeneity: $\chi^2_8 = 2.49$; p=0.96			*p = 0.3*

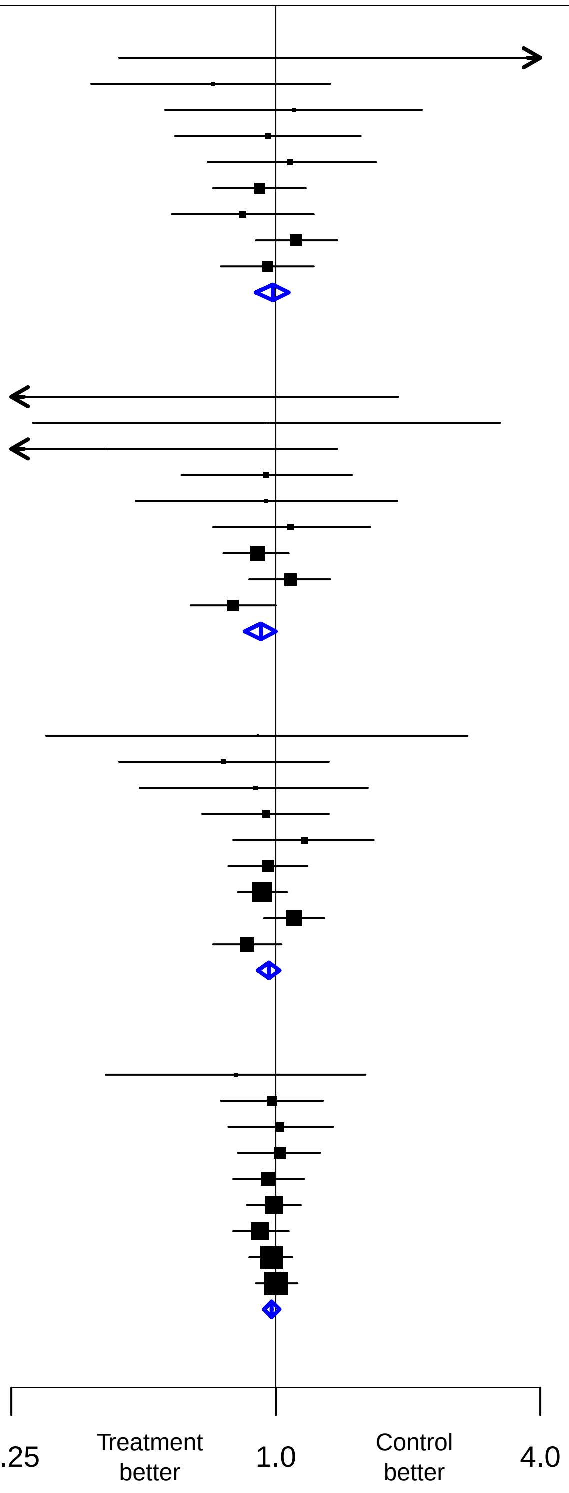

■ 99% CI
◇ 95% CI

© 2018 Aung T et al. JAMA Cardiol.

eFigure4: Effects of omega−3 fatty acids on risk of non−fatal myocardial infarction and stroke in SU.FOL.OM3, by analysis method

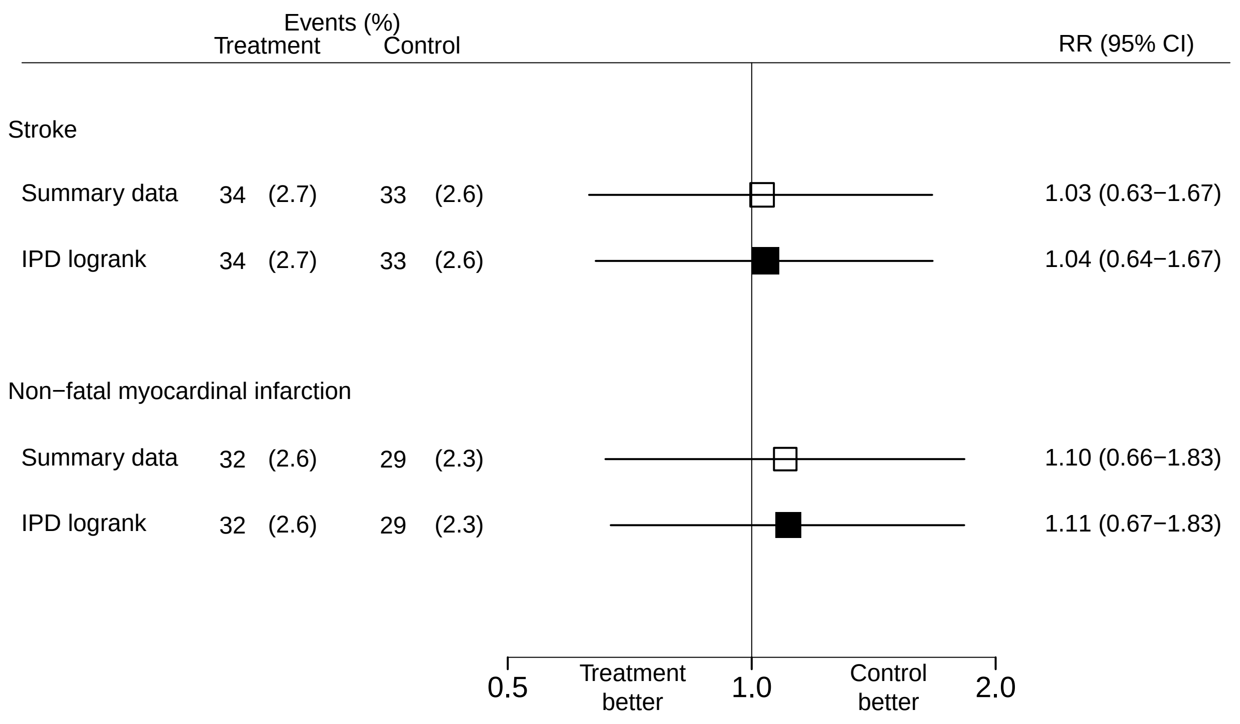

© 2018 Aung T et al. JAMA Cardiol.

eFigure 5: Effects of omega−3 fatty acids on total mortality, by trial

Trial	Events (%) Treatment		Events (%) Control		RR (CI)
DOIT	14	(5.0)	24	(8.5)	0.57 (0.24−1.35)
AREDS−2	30	(1.4)	27	(1.3)	1.06 (0.54−2.12)
SU−FOL−OM3	72	(5.7)	69	(5.5)	1.04 (0.67−1.63)
JELIS	286	(3.1)	265	(2.8)	1.08 (0.86−1.35)
ALPHA OMEGA	186	(7.7)	184	(7.6)	1.02 (0.78−1.36)
OMEGA	88	(4.6)	70	(3.7)	1.25 (0.82−1.90)
R&P	348	(5.6)	337	(5.4)	1.04 (0.85−1.27)
GISSI−HF	955	(27.3)	1014	(29.1)	0.92 (0.80−1.05)
ORIGIN	951	(15.1)	964	(15.4)	0.98 (0.86−1.11)
GISSI−P	477	(8.4)	554	(9.8)	0.85 (0.72−1.00)
ALL	3407	(8.7)	3508	(9.0)	0.96 (0.92−1.01)

Heterogeneity: $\chi^2_9 = 13.28$; p=0.15

p = 0.16

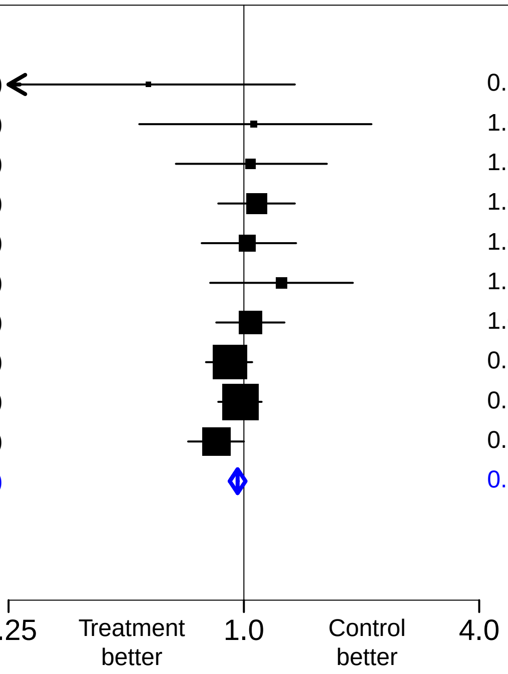

■ 99% CI
◇ 95% CI

© 2018 Aung T et al. JAMA Cardiol.
